# Revealing Three-Dimensional Printing Technology Advances for Oral Drug Delivery: Application to Central-Nervous-System-Related Diseases

**DOI:** 10.3390/pharmaceutics17040445

**Published:** 2025-03-31

**Authors:** Samir I. Paipa-Jabre-Cantu, Marisela Rodriguez-Salvador, Pedro F. Castillo-Valdez

**Affiliations:** Tecnologico de Monterrey, Monterrey 64700, Nuevo León, Mexico; a00818390@tec.mx (S.I.P.-J.-C.); a01318528@tec.mx (P.F.C.-V.)

**Keywords:** 3D printing, oral drug delivery, central nervous system

## Abstract

**Background/Objectives.** Central nervous system (CNS)-related diseases such as Alzheimer’s and Parkinson’s, Attention Deficit Hyperactive Disorder (ADHD), stroke, epilepsy, and migraines are leading causes of morbidity and disability worldwide. New solutions for drug delivery are increasingly needed. In this context, three-dimensional (3D) printing technology has introduced innovative alternatives to produce more efficient medicines with diverse features, patterns, and consistencies, particularly oral medications. Even though research in this area is growing rapidly, no study has thoroughly analyzed 3D printing oral drug delivery progress for the CNS. To fill this gap this study pursues to determine a technological landscape in this field. **Methods.** For this aim, a Competitive Technology Intelligence (CTI) methodology was applied, examining 747 publications from 1 January 2019 to 20 May 2024 published in the Scopus database. **Results.** The main advances identified comprise six categories: 3D printing techniques, characteristics and applications, materials, design factors, user acceptance, and quality processes. FDM was identified as the main technique for pharmaceutical use. The main applications include pills, polypills, caplets, gel caps, multitablets, orodispersible films, and tablets, featuring external patterns and internal structures with one or more active substances. Insights show that the most utilized materials are thermoplastic polymers like PLA, PVA, PCL, ABS, and HIPS. A novel design factor involves release patterns using compartments of varying thicknesses and volumes in the core. Additionally, advances in specialized software have enabled the creation of highly complex designs. In the user acceptance category, oral drugs dosages are tailored to the specific needs and preferences of neurological patients. Finally, for the quality aspect, the precision in Active Pharmaceutical Ingredient (API) dosage and controlled-release mechanisms are critical, given the narrow margin between therapeutic doses and toxicity for CNS diseases. **Conclusions.** Revealing these advancements in 3D printing for oral drug delivery allows researchers, academics, and decision-makers to identify opportunities and allocate resources efficiently, promising enhanced oral medicaments for the health and well-being of individuals suffering from CNS disorders.

## 1. Introduction

This study starts exploring the role of 3D printing in pharmaceutical manufacturing, particularly in the development of personalized oral drug delivery systems for central nervous system (CNS) diseases. By enabling precise dosage control and tailored formulations, 3D printing addresses key challenges in conventional drug production.

### 1.1. Context

Three-dimensional printing technology has revolutionized various sectors of the biomedical industry. It is a highly flexible manufacturing method with the potential to significantly innovate drug administration in living biological systems [1]. This technology has facilitated the creation of drug delivery systems, particularly in pharmaceuticals, where its application to traditional diseases has been explored [2,3]. Various administration routes exist for medications, including oral, nasal, vaginal, and rectal, with the oral route being the most effective in terms of patient adherence to treatments across all age ranges [4].

Drugs are prescribed according to different ages, from newborn to elderly [5]. During their lives, people face different challenges by taking medicines and adhering to their treatments. As individuals transition to geriatric ages, they often experience difficulties in swallowing medicines, memory impairment, cognitive decline, vision loss, and reduced dexterity, affecting their medical treatments. On the other hand, many patients require complex polypharmacy regimens that are difficult to follow with traditional medications. Individuals with neurological diseases that cause visual impairments, approximately 1.5 billion people worldwide, encounter challenges in distinguishing the name, dosage, and expiration dates of medications, which are usually printed graphically on conventional packaging [6].

These factors present a significant opportunity for the development of products that cater to the diverse needs of this population. With 3D printing, patients of all ages are increasingly favoring oral medications in the form of orodispersible films, gummies, and sublingual orodispersible tablets for the ease of self-administration and the perception of safer digestion and effectiveness [7]. Personalizing doses of active substances is crucial to achieving therapeutic effects, and the treatment of CNS diseases has a main role in the global market.

CNS-related diseases are a leading cause of morbidity and disability worldwide. As detailed in ‘Global, Regional and National Burden of Disorders Affecting the Nervous System’ (*The Lancet*, 2024), between 1990 and 2021, these diseases affected 43% of the global population—approximately 3.4 billion people—with 443 million experiencing a poor quality of life [8]. Additionally, patients represent a significant global market share, as will be described in the next section. Conditions such as stroke, neonatal hypoxic ischemic encephalopathy, migraine, dementia, diabetic neuropathy, meningitis, epilepsy, and neurological complication in preterm birth and being on the autism spectrum were identified as the most prevalent [9].

Oral medications are fundamental for the control of these diseases as this type of drug delivery has been recognized as the fastest, simplest, and most comfortable route, often outperforming other methods [10]. The process used to fabricate medications is determined by several factors including the characteristics of the APIs and the specific requirements of the excipients, aiming to achieve the desired therapeutic effects. The effectiveness of the Active Pharmaceutical Ingredient (API) in oral medications depends on several factors, including the physical state of the APIs (e.g., pulverized solid, semi-solid, or gel), the route of entry, and transportation, distribution, and absorption within the gastrointestinal surface, which also influence the drug’s performance [11].

The conventional production of oral medications for CNS diseases includes methods such as direct compression, dry granulation, and wet granulation. These methods use raw materials such as APIs, excipients, diluents, disintegrants, binders like povidone (PVP), lubricants, and coatings like hydroxypropyl methylcellulose (HPMC) [12].

The three-dimensional printing of oral medications for CNS diseases offers numerous advantages over conventional manufacturing methods, such as those previously discussed. These advantages include greater customization and precision, and the ability to design medications with advanced properties, as clinical trials have shown [13]. Traditional methods face limitations; for example, direct compression can only be used with APIs in powder form that have good flowability and compactability, with limited capacity for the customization of shapes and release profiles. Meanwhile, dry granulation allows the production of products with lower homogeneity compared to other conventional methods, making it less effective for formulating complex release profiles—an area where 3D printing excels. Finally, wet granulation is costly, time consuming, and unsuitable for ingredients sensitive to moisture or heat [14], where 3D printing provides unique solutions.

For CNS medications, 3D printing provides significant advantages, as therapies often need to be tailored to individual patient needs and optimized for controlled drug release to maximize efficacy while minimizing adverse effects [15]. Moreover, 3D printing addresses the high costs associated with CNS disease treatments. By enabling smaller doses and faster production times, and reducing material waste, this technology significantly lowers manufacturing expenses. On-demand production further cuts costs by eliminating the need for large-scale manufacturing, extensive inventory storage, and costly logistics such as transportation and cold chain requirements for temperature-sensitive medications. These benefits collectively position 3D printing as a cost-effective alternative for CNS treatment production [4].

In particular, this technology offers an effective means of oral drug delivery, enabling the production of complex designs [16]. It allows the creation of oral vehicles in various forms, such as orodispersal films, pills, polypills, and multi-compartmental tablets, that optimize the effectiveness of the active substances they contain. The goal is to reduce patient mortality and morbidity, slow the progression of symptoms, and decrease therapeutic failures caused by poor adherence to medications [17]. This issue is particularly relevant because oral treatments involve conventional dosing, as presented in the *Manual of Adherence to Chronic Treatments* by the Pan American Health Organization, in the section on neurological medications and the lack of effectiveness of neurological medications [18]. Despite considerable research efforts in 3D printing for oral drug delivery [19,20], no study is available regarding advances around 3D printing for oral drug delivery for CNS conditions. Current research focuses on the applications of 3D printing in healthcare [1,2,7], 3D bioprinting for drug delivery [10,11,19], and 3D printing technologies for pharmaceutical manufacturing [5,11,13,14,16,17]. To fill this gap, a Competitive Technology Intelligence (CTI) methodology was implemented to reveal the 3D printing advances for oral drug delivery in CNS-related diseases, aiming to support health professionals, researchers, and scholars in the technology decision-making process.

CTI is a strategic approach based on a continuous process focused on identifying technological trends and opportunities in a specific field, offering insights for decision making in technology, innovation, product design, research, and market analysis [21]. CTI stands apart from conventional information methods by adopting a proactive approach, anticipating trends, and guiding the early adoption of transformative technologies. It provides a comprehensive, global analysis of technological, regulatory, and market landscapes, integrating multidisciplinary insights to drive innovation. Unlike traditional methods with lengthy evaluation cycles and standardized practices, CTI contributes to accelerate technology adoption and emphasizes personalization and adaptability, which are critical for addressing the unique complexities of CNS medications.

In this study, a CTI methodology comprising eight steps considering the approach of Rodriguez-Salvador and Castillo-Valdez (2021) [21] was applied as shown in a subsequent section.

### 1.2. Global Market for Personalized Neurological Drugs via 3D Printing

The global market for 3D-printed pharmaceuticals is experiencing significant growth, propelled by several factors, such as the use of new manufacturing technologies to achieve a more integrated healthcare ecosystem [22]. These include increased government investment in 3D printing technology, venture capital funding for startups in the field, and the pharmaceutical industry’s expanding adoption of this technology. An Organization for Economic Co-operation and Development (OECD) analysis highlights an increase in per capita government health spending in industrialized nations [23]. Personalized medicines made possible by 3D printing represent a radical shift in healthcare treatments and production processes, offering more agile and adaptable manufacturing methods. Additionally, a recent study found that approximately 63% of pharmaceutical companies are considering investing in 3D printing, with the number of professionals utilizing this technology having tripled since 2017 [24]. Currently, five pharmaceutical companies are leading the production of 3D printing technologies for pharmaceuticals: Aprecia Pharmaceuticals (Langhorne, PA, USA) pioneered the first FDA-approved 3D printing platform in 2015 for commercial-scale drug production, using its Zip Dose technology to develop Spritam, a 1000 mg levetiracetam tablet that rapidly dissolves with a sip of water. FabRx (London, UK) introduced the M3DIMAKER 3D printer, the first pharmaceutical printer for personalized medicine, capable of producing “printlets” with Braille text or dotted patterns for visually impaired patients. Merck (Darmstadt, Germany), in partnership with Additive Manufacturing Customized Machines (AMCM), a division of Electrical Optical Systems (EOS), focuses on industrial applications of 3D printing for large-scale drug manufacturing. Triastek (Nanjing, China), with 41 patents for 3D-printed pharmaceutical applications, developed an FDA-accepted 3DMED platform, enabling tablets with diverse shapes and geometries for controlled API release. The company also introduced chronotherapeutic drugs for rheumatoid arthritis and neurovascular disorders and collaborated with Eli Lilly to produce 3D-printed polypills with enhanced bioavailability and targeted release for the central nervous system. GlaxoSmithKline (London, UK), in collaboration with the University of Nottingham, has pioneered commercial-scale inkjet 3D printing with ultraviolet curing for solid medications, becoming the first in 2017 to 3D print ropinirole tablets for Parkinson’s disease while also exploring curable API inks for 3D printing [25].

Financial backing has played a crucial role in driving this market expansion, with countries such as the United States, Canada, Germany, the United Kingdom, France, Austria, Japan, India, China, and South Korea offering incentives to support investments in advanced digital technologies, including 3D printing [26,27].

In recent years, there has been a growing interest in personalized medicine, with 3D printing playing an increasingly vital role. This technology enables tailored dosages, enhances medication retention rates, and facilitates easier swallowing through various medication forms [28].

## 2. Methodology

**Figure 1:**
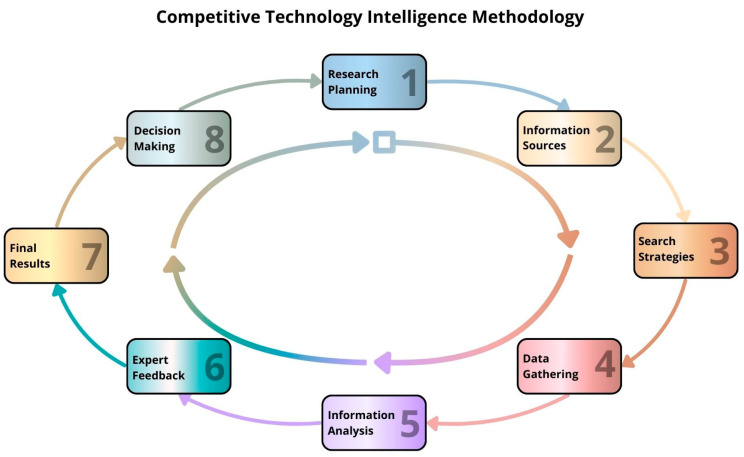
Competitive Technology Intelligence methodology. Adapted from Rodriguez-Salvador and Castillo-Valdez (2021) [21].

This study applied a CTI methodology based on the approach of Rodriguez-Salvador and Castillo-Valdez [21], which comprises eight steps, as follows: (1) research planning, (2) identification of data sources, (3) design of search strategies, (4) data collection, (5) information analysis, (6) expert feedback, (7) validation and final results, and (8) decision making (Figure 1).

For the first step of this study, a work plan was developed that included specific objectives, gathering dates, task distribution, and people’s roles to ensure organized execution. The research relied on high-prestige databases, specifically Scopus, focusing on the most recent years available at the time of the investigation. In this study, scientific publications were considered, as they provide peer-reviewed, scientifically validated insights into the latest advancements and research findings. Additional metrics included the expertise and reputation of experts, the alignment of publications with the study’s objectives, and the inclusion of peer-reviewed scientific journals. According to Scopus, scientific journals are defined as periodic publications containing original articles on academic and scientific topics, reviewed by experts in the field, with the aim of disseminating research results and advancing knowledge in various areas.

For the second step, the information sources were identified, with the primary source being the expert’s determination. A distinguished pediatric neurologist, professor of neurology for general practitioners, and director of the academic and research program in postgraduate studies in pediatric neurology at the Universidad Autónoma de Nuevo León (UANL) participated in this study. This professional holds a highly prestigious international reputation in the field. Scopus, which is a prestigious database that includes more than 46,000 peer-reviewed journals and 20.5 million open-access records, was selected as the secondary source [29]. As the third step, the search strategy was designed, covering the period from 1 January 2019 to 20 May 2024, when the gathering activity ended. Experts in the field of neurological diseases were consulted, and an initial review of the literature was conducted to identify a first research topic. 

The research topics were identified through primary consultation with experts in the field of neurological diseases, followed by an initial review of the literature.

Based on this, keywords were determined to guide the search process. Additionally, Scopus filtering options were utilized to strategically categorize the sources for analysis, aligning with the methodological design. The publications were organized manually by specific topics, with a focus on identifying high-impact studies, recent publications, original research, and systematic reviews to facilitate further analysis.

The fourth step of the methodology focused on the gathering process. Relevant terms related to 3D printing for oral drug delivery systems and the CNS were identified, including “3D printing”, “personalized medicine”, “neurology”, “neurological”, “oral”, “pediatric”, “geriatric”, “drug administration”, and “release methods”. Pediatric and geriatric keywords were selected since, from all age ranges, these groups could benefit the most from 3D printing for oral drug delivery. A total of 718 publications were collected and manually reviewed to refine the results as follows: (1) review articles had to be excluded, (2) the topic had to relate to techniques, applications, materials, or quality of 3D printing, and (3) the focus had to be on oral drug delivery of 3D-printed medications for the CNS. After these validations, the number of documents was reduced to 437. Based on the information obtained, an analysis was performed in the fifth step aiming to classify the documents retrieved and identify the most relevant technological trends. In total, 93 publications were detected, and, according to the results obtained and previous research [20], they were categorized into areas such as printing techniques, applications, materials, design factors, user acceptance, and quality processes. The expert’s feedback in the sixth step was obtained throughout the different steps of the CTI methodology. In the seventh step, insights were obtained, which are shown in the Section 3. Finally, stakeholders, including experts, researchers, and decision-makers, can analyze the trends to make well-informed decisions in the eighth step, aligning with the study’s objectives.

## 3. Results and Discussion

The results obtained show that 3D printing represents an innovative option for oral drug delivery in CNS diseases. First, they indicate that this technology overcomes the lack of precision in drug release by enabling the design of customized pharmaceutical forms that guide the dissolution and absorption to specific areas of the gastrointestinal tract, optimizing the bioavailability of APIs. Second, it addresses low adherence in neurological patients through tailored presentations, such as orodispersible tablets or prolonged-release formulations, which reduce the frequency of intake and enhance the patient experience. Finally, the results show that 3D printing surpasses the limitations of conventional treatments by offering versatility in incorporating one or more APIs with personalized release patterns, increasing therapeutic efficacy and improving patients’ quality of life. These findings underscore the importance of 3D printing in overcoming current barriers in the treatment of neurological diseases.

**Figure 2:**
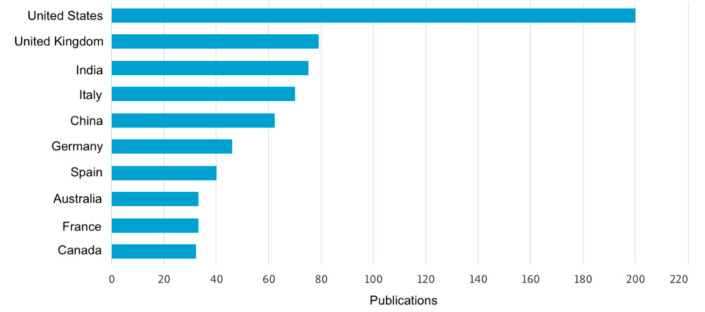
Publication contribution by country or territory.

**Figure 3:**
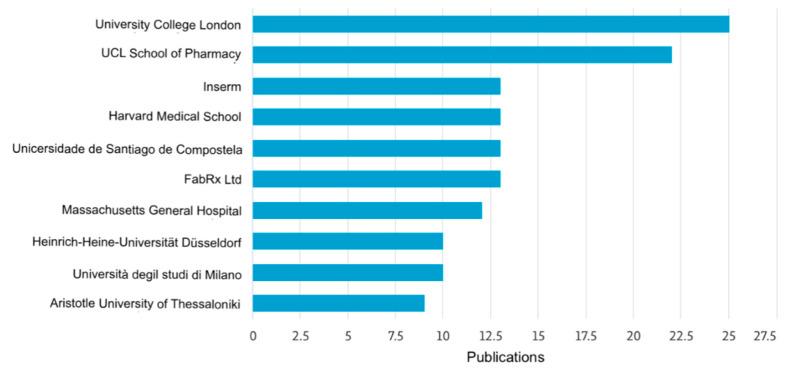
Affiliations with significant contributions.

**Figure 4:**
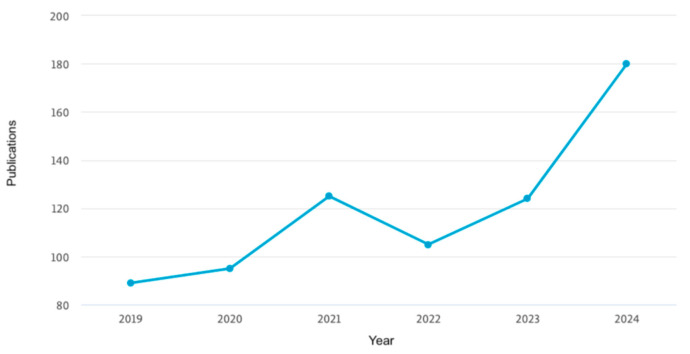
Yearly progression of publications. (The analysis concluded on 20 May 2024, so the data for 2024 cannot be considered as representing a full year).

The following figures provide insights based on key metrics and trends related to the scientific research on 3D printing for oral drug delivery in CNS diseases. They illustrate the geographic contribution of publications (Figure 2), affiliations with significant contributions (Figure 3), and the yearly progression of publications within the specified timeframe (Figure 4). These data serve as the foundation for understanding the current research landscape and the growing importance of this field.

The analysis of the data reveals significant growth in the number of publications related to 3D printing for oral drug delivery in CNS diseases over time, with a sharp upward trend observed between 2019 and 2024. In 2019, there were 89 publications, which steadily increased to 124 in 2023 and reached 180 by 20 May 2024, marking an overall increase of approximately 102% since 2019. However, it is important to note that the 2024 data only represent five months, suggesting that the full year could potentially exceed this number. In terms of contributions by country, the United States leads with the highest number of publications, significantly outpacing other countries, followed by the United Kingdom, India, Italy, and China, which, together, represent key contributors to the global research efforts in this domain. Regarding institutional affiliations, University College London stands out as the top contributor, followed by UCL School of Pharmacy, Inserm, and Harvard Medical School. Additionally, other prominent institutions include the Universidad de Santiago de Compostela and FabRx Ltd. Overall, this growth reflects the increasing importance of this research area and the collaborative efforts of leading countries and institutions to advance knowledge and innovation of 3D printing for oral drug delivery for the CNS.

Based on the analyzed information regarding CNS oral drugs, the following six categories were identified: 3D printing techniques, applications, materials, design factors, user acceptance, and quality processes, which are described below (Table 1) and will be analyzed in further sections.

### 3.1. Three-Dimensional Printing Techniques for Central Nervous System (CNS) Oral Drugs

Three-dimensional printing technologies such as Fused Deposition Modeling (FDM), Semi-Solid Extrusion (SSE), Stereolithography (SLA), Digital Light Processing (DLP), Selective Laser Sintering (SLS), and Binder Jetting (BJ) are being used in the development of Neurological Drug Administration Systems (NDDS), ranging from neuromodulatory molecules to vitamin and mineral supplements. These systems include oral vehicles capable of the controlled release of mono- or multidrugs, such as caffeine Oral Dispersible Films (ODF) for migraine headaches, which provide immediate release, and methylphenidate, which uses prolonged disintegration through pulses at various frequencies and pre-established times.

These oral delivery vehicles improve the distribution of the APIs they contain to meet the therapeutic needs of patients with CNS diseases, as described by [30]. This process is based on the digitally controlled layer-by-layer deposition of materials to form various formulations and geometries [31].

The production cost of 3D-printed medications for the CNS is highly variable as it depends on the specific disease being targeted, which dictates both the printing technique and the materials utilized. Moreover, the selection of methods and materials is significantly influenced by the API being used. The properties of the API, such as its sensitivity to heat, solubility, and stability, play a critical role in determining the appropriate printing technique and compatible materials. For instance, certain APIs may require specific polymers or excipients to ensure compatibility and efficacy, while the choice of printing technique must align with the API’s physical and chemical characteristics and the rate of disintegration of the oral vehicle (orodispersal films, pills, polypills, tablets, etc.), which is absorbed in the digestive system and travels through the circulatory system to the brain. This intricate relationship between the API’s attributes and production constraints makes the selection process highly contextual and necessitates a careful evaluation of all variables. Consequently, establishing a generalized cost is not feasible due to these complex and interdependent factors [12].

Experts have played a key role in identifying the commercial applications of APIs targeting CNS diseases administered orally and manufactured through 3D printing. Their expertise enabled the evaluation of specific therapeutic needs, the advantages of dose customization, and the potential benefits of this technology in terms of efficacy, safety, and accessibility.

These printing techniques are described below. While the shapes that these techniques can create can differ, to facilitate the visualization of the techniques, only capsule-based shapes are shown.

#### 3.1.1. Fused Deposition Modeling (FDM) Technique

**Figure 5:**
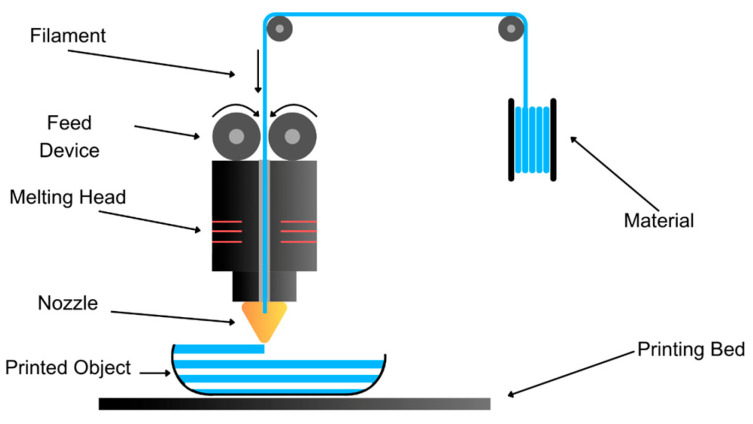
Schematic representation of the FDM technique illustrating the layer-by-layer deposition process of thermoplastic material to create complex 3D structures.

This process is part of material extrusion printing, where the material is distributed through a heated nozzle. Together with the movement of the printing head, the required structure is generated by extruding the filament onto a platform in a controlled manner, with xy movements forming each layer, and a vertical movement along the *z*-axis to define the dimensions (Figure 5). In this process, one or more nozzles can be utilized, enabling the simultaneous printing of different active ingredients. This is particularly useful for neurological medications, where multiple drugs with different physical structures need to be administered [32]. Advances in these processes involve the rapid integration of technology, such as the recent use of microfluidic chips. These chips can provide support and malleability to the molten material for extrusion and layer deposition [33].

FDM has advantages such as lower cost and greater homogeneity of the active substance, and does not need post-processing. However, it has disadvantages, including the high printing temperatures required, which are not suitable for thermolabile active substances that may need preprocessing. This technique has been used to print various drugs, such as cinnarizine for central vertigo; haloperidol as an antipsychotic; theophylline as a xanthine stimulator of the central nervous system; pregabalin and carbamazepine orodispersibles for epilepsy and central pain; rufinamide for Lennox–Gastaut (SLG) epileptic syndrome [34]; aripiprazole as an orodispersible for bipolar disorder, AHDA, and Tourette syndrome [35,36,37]; olanzapine as an antipsychotic and prednisolone as a steroid [38]; rasagiline mesylate, levodopa, benserazide, and pramipexole for Parkinson’s disease [30]; zolpidem and caffeine–melatonin for insomnia [39]; venlafaxine and mirtazapine orodispersibles as antidepressants [40,41]; paroxetine–magnesium stearate–dicalcium dehydrate phosphate as a selective serotonin reuptake inhibitor, used as an antidepressant [14]; praziquantel for cerebral cysticercosis; and dopamine for abnormal movements [30].

#### 3.1.2. Semi-Solid Extrusion (SSE) Technique

**Figure 6:**
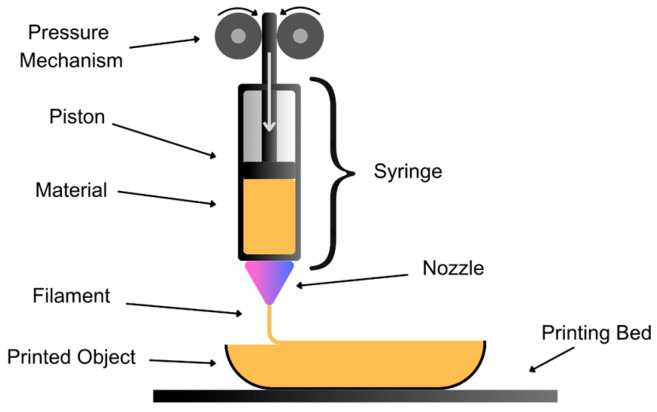
Schematic representation of the SSE technique showcasing the extrusion of semi-solid formulations through a syringe-based system to create precise 3D-printed structures.

Three-dimensional printing by SSE is a technique based on the extrusion of FDM material (Figure 6). It offers several advantages; for example, it is performed at room temperature—thereby avoiding the risk of heat-induced degradation of the active substance—using viscous rather than molten materials and capturing a high load of the active substance. It also demonstrates suitable behavior for the printing of multidrug and polypill systems. However, its limitations include the print resolution being dependent on the size of the printing nozzle, the need for a post-processing drying stage, and the rheological properties of the polymer, which affect the structural integrity of the prints.

SSE printing is performed with semi-solid materials, such as pastes or gels, which are extruded using a pneumatic, mechanical, or solenoid piston through the head of a syringe. The sequential layers are deposited and then solidify through evaporation at room temperature [42].

The semi-solid materials used include polymers, with active ingredients and excipients integrated into a paste that is extruded through the syringe. These polymers must exhibit good mechanical resistance and rapid gelation to withstand the superposition of layers. It is important to consider the rheological properties of the materials, as the viscosity needs to decrease rapidly at the syringe and then recover immediately after the pressure is released [43].

Oral vehicles produced by SSE printing contain APIs such as propranolol for treating migraine headaches, theophylline as a stimulant of the central nervous system in neonates, carbamazepine [44,45], phenytoin, lamotrigine, and levetiracetam [43,46], and gabapentin and pregabalin [47] as antiepileptics. Additionally, mirtazapine [41], zolpidem [48,49], a sleep inducer and neuromodulator for neuropathic pain, olanzapine [50], venlafaxine [40], and paroxetine [14] are also used. A notable application is the production of warfarin ODF for patients with neurovascular diseases who have difficulty swallowing [51].

#### 3.1.3. Stereolithography (SLA) and Digital Light Processing (DLP) Techniques

**Figure 7:**
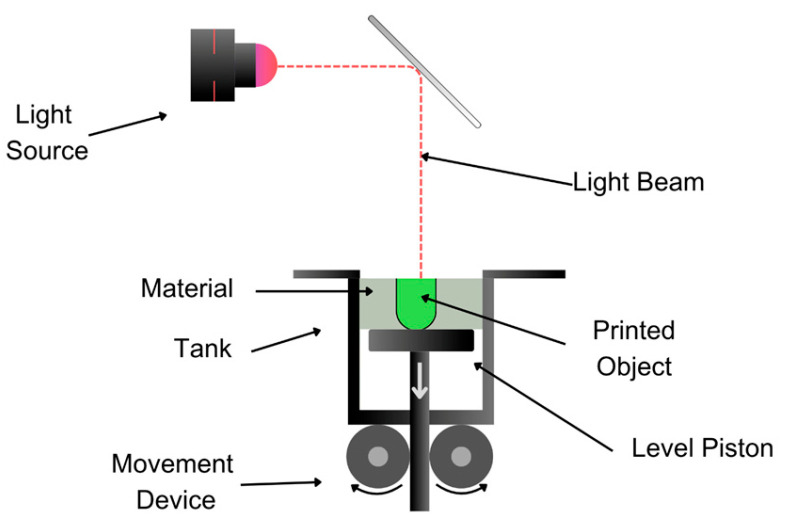
Schematic illustration of the SLA technique demonstrating the layer-by-layer curing process of photosensitive resin using a UV laser.

**Figure 8:**
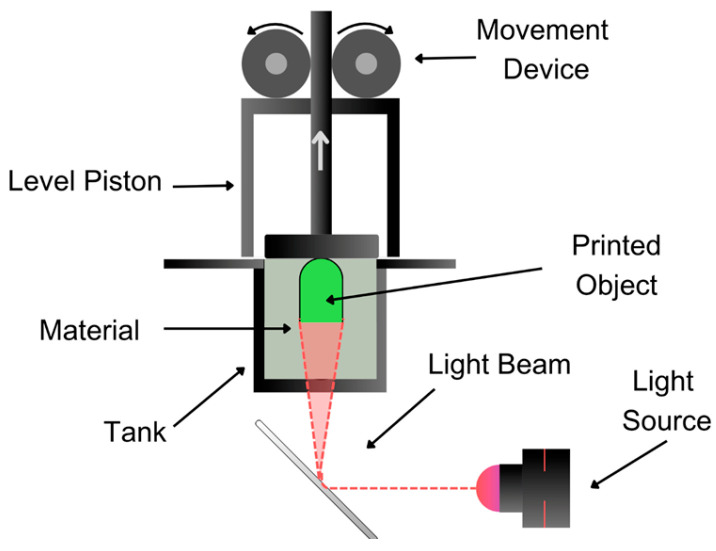
Diagram of the DLP technique showing the projection of light patterns onto a vat of photopolymer resin.

These processes involve very high precision and resolution, using liquid photopolymeric resins that selectively solidify through light-activated polymerization reactions. In the case of SLA, ultraviolet (UV) light is used (Figure 7), while DLP utilizes a digital projection screen that projects the image onto the platform (Figure 8). This type of process was applied by authors such as Martínez et al. [6] in the generation of therapeutic combinations through multiple personalized layers. With varying drug concentrations and geometric shapes, more versatile drug formulations can be achieved [52], exhibiting adequate dissolution and release profiles [53]. Additionally, the drug release profile can be further modulated by adjusting factors such as the particle size, geometric structure, surface-area-to-volume ratio, or the infill density of the printed tablets. For instance, an 8 mm printed tablet with a 90% infill demonstrated a controlled release over a period ranging from 40 to 852 min [54]. On the other hand, this technique has the disadvantage of generating significant material waste due to the nature of the process, with typically 10–50% of the photopolymer resin remaining unused because of factors such as object design, build area size, and resin recovery system efficiency. This issue is especially costly in pharmaceutical applications, where specialized, high-purity resins for drug-loaded formulations are required [15].

APIs such as paracetamol, naproxen, caffeine, aspirin, prednisolone, and chloramphenicol in one polypill are used for central nervous system bacterial infection [55,56], caffeine–melatonin for sleep regulation [57], theophylline as a stimulant of the central nervous system for sleep regulation [58], paracetamol for headaches and non-oncological pain [59], carbamazepine as an antiepileptic [45], roripinol for Parkinson’s disease [60], and methylphenidate for Attention Deficit and Hyperactivity Disease [61].

#### 3.1.4. Selective Laser Sintering (SLS) Technique

**Figure 9:**
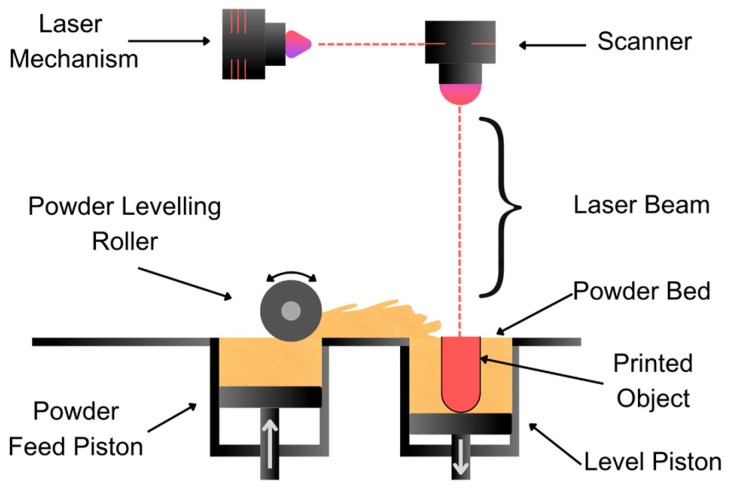
Schematic representation of the SLS technique depicting the use of a high-powered laser to fuse powdered material layer by layer.

This process, known as SLS, involves the use of a powder bed, where the active ingredients and excipients are combined. A laser selectively melts areas of the bed to form the superimposed layers (Figure 9). Permeable structures, with porosity ranging from 10% to 70%, are highly beneficial for controlling the drug release rate in 3D-printed medicines for CNS disorders. Higher porosity (50–70%) enhances drug diffusion and allows rapid release for acute conditions like migraines or seizures, while lower porosity (10–30%) slows release, making it ideal for sustained therapies in chronic CNS conditions like Parkinson’s or depression. Additionally, porosity increases surface area for better dissolution of poorly soluble CNS drugs, supports personalized dosing, reduces dosing frequency, and improves bioavailability, offering significant adaptability for tailored CNS treatments [62,63].

In this technique, the pharmaceutical ink consists of a powder containing the API and necessary excipients. A layer of this powder is placed on a platform, where the laser light beam heats it slightly below its melting point, initiating a sintering process in which the particles fuse and solidify [64].

The unmelted powder acts as a support for the geometric structure during the process, eliminating the need for additional support materials. Overlapping layers, ranging in size from 50 to 200 microns, are printed, and a roller evenly distributes the powder mixture after each layer is printed, ensuring a homogeneous process. Once printing is completed, a cooling process is required in the chamber. The object is then removed and allowed to rest for a predetermined time to acquire its mechanical properties, preventing deformation. After this period, the excess powder, which contains APIs and excipients, is discarded, representing an economic limitation [65]. This process generates a useful porous structure for drugs requiring prolonged release [66]. Recent research has focused on the use of magnetic nanoparticles as an alternative to excipients to facilitate sintering in the printing of oral tablets [67]. Paracetamol, a first-line pain reliever for headaches [62], and ibuprofen are used for headaches and chronic neuropathic pain [68]. Ondansetron, an antiemetic, is used to prevent vomiting and nausea induced by chemotherapy, radiotherapy, and neoplasia in the CNS [69]. These are produced using this printing method.

#### 3.1.5. Binder Jetting (BJ) Technique

**Figure 10:**
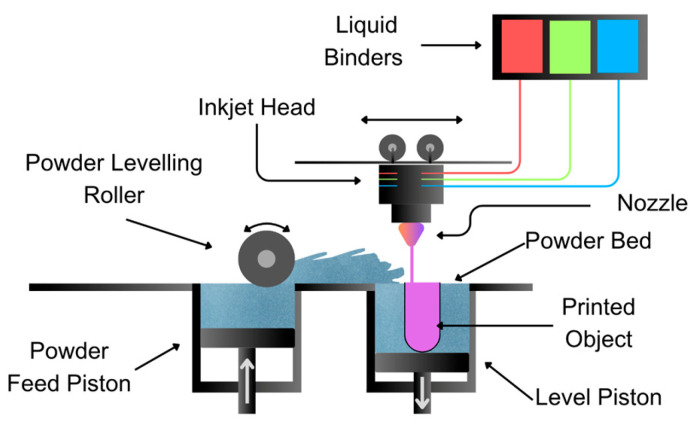
Illustration of the BJ method showcasing the process of selectively depositing a liquid binder onto a bed of powdered material.

In this process, a binder is selectively dripped onto a bed of powder containing active ingredients and excipients, which subsequently solidify (Figure 10). The advantage of this method is that it operates at room temperature, though it requires a post-processing stage. However, there is instability in the degree of porosity in the final product.

As the first 3D-printed vehicle for neurological use to reach the market at an industrial level, Aprecia Laboratories introduced Spritam^®^ [70], a 1000 mg [71] oral dispersible tablet used as an antiepileptic medication. These tablets are orodispersible and printed with a cover and a direct-release tab using an automatic zipper dispensing mounting system. The tablet has a disintegration time of less than one minute, facilitating easier swallowing and a rapid onset of action [72]. In addition to levetiracetam, the method is used to print levetiracetam–pyridoxine [46] and the pain relievers paracetamol, diclofenac, and acetaminophen [73].

According to the analysis, it was identified that, to date, the FDM printing technique has demonstrated the most favorable pharmacological results for the treatment of CNS diseases, followed by SLA. Future efforts should continue to advance the FDM process, as it offers several advantages, including lower cost and greater homogeneity of the active substance, and does not need post-processing. However, its limitations, such as the high printing temperatures required and unsuitability for thermolabile active substances that may require preprocessing, must also be addressed to further optimize its applicability.

### 3.2. Characteristics and Applications

Traditional oral medications often rely on standard doses of active substances in capsule form, which may not align with individual patient needs. Factors such as disease state, age, sex, weight, and metabolism can lead to underdosing or overdosing. This is particularly important for neurological drugs with a narrow therapeutic margin. Even minor variations in these factors can significantly impact drug effectiveness and safety. Three-dimensional printing addresses these limitations by allowing precise customization of active ingredients, controlled-release mechanisms, and dosage forms tailored to patient-specific factors. This technology enables the production of a wide variety of shapes and dimensions from thin and rapidly dissolving matrices with mono- or multidrug release profiles, producing complex customizable geometries, such as cylindrical tablets; spherical polypills; flexible gummies; and unique films, which are currently highly in demand [74,75]. Cylindrical tablets are regularly used for sustained-release formulations, while films are primarily designed for rapid dissolution and absorption. By controlling geometry, internal structures, and polymer composition, 3D printing facilitates targeted drug release, improved bioavailability [56], and even dual-release mechanisms that enhance drug dispersion across different times and areas of the digestive system [76], characteristics that contribute to medication reaching the desired site of action more efficiently. In addition, 3D printing provides oral vehicles exhibiting resistance to premature disintegration caused by variations in gastrointestinal pH and gastric emptying speed. This distinctive property favors drug release in the optimal area for intestinal absorption, which is crucial for successful therapeutic effects [77,78]. For instance, orodispersible films can be engineered with tissue patterns and specific thicknesses to disintegrate instantly in the oral cavity upon contact with salivary enzymes like ptyalin and salivary amylase. This enables rapid absorption of neurological medications such as triptans, used in treating acute migraine symptoms [57]. These are the valuable characteristics of 3D printing that favor optimal API dosage delivery within the CNS. They are tailored to patient needs and cause the remission or delay of pathological activity, as neurophysiological studies such as electroencephalograms have shown [79,80], enhancing treatment efficacy, minimizing adverse effects, and improving patient adherence [38,81]. Personalized dosing can also reduce the frequency of therapeutic failures and hospitalizations caused by medication-related issues, ultimately improving long-term patient outcomes.

In addition, the technology’s versatility extends to creating internal multi-compartment structures that enable the delivery of multiple drugs or varying doses of the same drug over time, as demonstrated with methylphenidate [82]. Variations in compartment thickness, polymer types, and process parameters can further influence release patterns, as seen with formulations such as levetiracetam. Additionally, combining active substances with different consistencies, such as caffeine and paracetamol, within a single oral vehicle exemplifies the broad capabilities of 3D printing. Adjusting filament composition, extrusion rates, printing angles, and orientations enables the production of predetermined shapes using technologies such as FDM. This design flexibility allows for complex drug release profiles that can accommodate circadian rhythms or specific pharmacokinetic requirements, offering significant advantages over traditional manufacturing methods.

The growing interest from both the scientific community and the pharmaceutical industry underscores the potential of 3D printing for developing oral drug delivery systems for neurological diseases. Notable applications include antiepileptics such as carbamazepine, phenytoin, lamotrigine, and levetiracetam; neuromodulators and antiepileptics such as gabapentin; sleep inducers such as zolpidem; and antidepressants such as mirtazapine, olanzapine, venlafaxine, and paroxetine. These medications, often used in chronic conditions, benefit greatly from tailored dosing and controlled-release features to reduce side effects and improve patient compliance. One of the most promising areas is the development of the 3D printing of polypills, caplets, gel caps, orodispersible films, and tablets with intricate external patterns and internal structures that contain one or more active substances. These advanced designs enable single or multiple active substance delivery in a controlled, patient-centric manner, thereby streamlining complex treatment regimens and potentially enhancing therapeutic efficacy across a wide range of neurological conditions.

### 3.3. Printing Materials

The use of materials to produce different consistencies of excipients is fundamental to innovation within 3D printing, particularly in the production of personalized oral medications, including the use of APIs for managing diseases of the central nervous system. In this context, 3D prints with soft consistencies, such as gel caps, orodispersible firms, or films with different 3D printing patterns, generate a precise seal with dimensional harmony, overcoming traditional production defects like surface coverage issues, defective sealing, or asymmetry, and reducing production costs [83].

These processes use thermoplastic polymers such as PLA, PVA, PCL, ABS, and HIPS, which exhibit versatile applications in pharmaceutical 3D printing. Initially used in neural conduit fabrication, these materials later advanced to the production of oral vehicles like hydrogels and are now being adapted for manufacturing gel capsules [84]. These capsules feature polymeric networks with high water absorption capacity, emphasizing their role in sustained drug release [57]. PLA is employed in implants, scaffolds, and drug delivery systems. Its thermoplastic properties enable processing via FDM, allowing tailored degradation rates for prolonged release. Conversely, PVA’s water solubility, biocompatibility, and mechanical enhancement properties make it suitable for oral caplets, tablet casings enabling zero-order release, and controlled-release shells [5]. Additionally, these polymers can form complex geometries, facilitating the design of nanotechnological medicines [85] to address specific delivery challenges [86].

During printing, the polymer filament has the ability to, if required, combine with the active ingredients of the medication, a process that can be carried out through immersion in a solution. The traditional process involves passive diffusion, where the filament is immersed in a solution saturated with the drug’s active ingredient, such as caffeine orodispersible films [79].

According to the analysis of the materials mentioned above, PLA and PVA are the most used in pharmaceutical production due to their characteristics, which are described below.

PLA is a natural, organic, biodegradable, and biocompatible polymer. However, it has hydrophobic properties, which is a biomedical disadvantage, as it poses difficulties in dissolution and disintegration, affecting the optimal release of active substances. It may also trigger inflammatory responses. PLA is used in venlafaxine production for treating depression [87]. PVA, on the other hand, is a hydrophilic, biodegradable, biocompatible, and non-toxic synthetic polymer, though it is only slightly soluble in ethanol and insoluble in organic solvents. It is used in pharmaceuticals due to its suitable viscosity and low melting temperature. However, its hygroscopicity can alter the release and action of the drug. PVA is used in medications such as haloperidol, pramipexole, levetiracetam, and aripiprazole [57].

Currently, polymer mixtures are used to create filaments necessary for printing oral vehicles for oral drugs such as levetiracetam and lamotrigine [11]. These mixtures enhance their rheological properties, enabling the printing of specific three-dimensional structures, thus providing stability to the composition of the active ingredients for optimal function and ensuring compliance with pharmacological quality standards.

### 3.4. Design Factors

Three-dimensional printing technology has transformed the development of personalized oral drug delivery systems by enabling the creation of internal structures tailored to specific therapeutic needs. Using Computer-Aided Design (CAD) files, this technology allows for precise control over the printing pattern and the inclusion of APIs. This flexibility facilitates the customization of dosage forms by adjusting parameters such as size, shape, amount, and type of API [88], providing highly targeted therapeutic solutions. Furthermore, these systems are valued for their ability to undergo structural modifications during manufacturing based on the APIs they contain [37], which enhances their adaptability to different medical applications.

**Figure 11:**
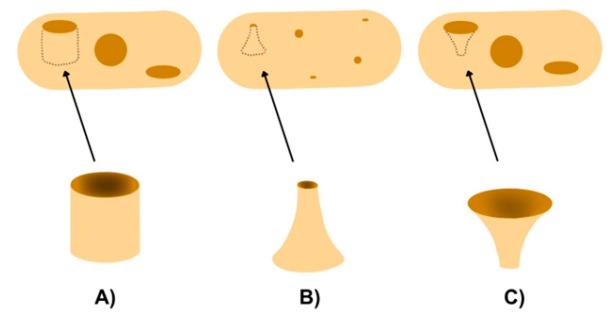
Three-dimensional-printed paracetamol tablets with varying geometries to achieve distinct release profiles: (**A**) cylindrical design, (**B**) horn-shaped structure, and (**C**) inverted horn geometry.

Innovations in 3D-printed oral drug delivery vehicles primarily focus on geometric patterns that improve patient ingestion and control the release of active substances. For example, research by Krueger et al. [89] highlights the use of diverse cavity shapes such as cylinders, horns, and inverted horns (Figure 11) to achieve distinct release profiles.

**Figure 12:**
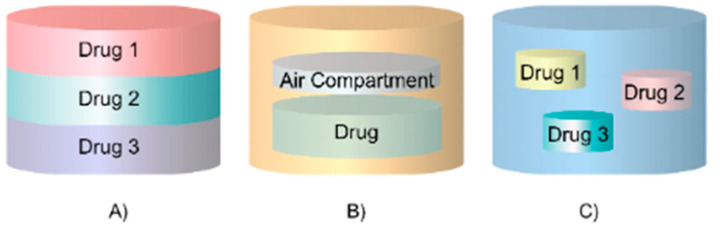
Three-dimensional-printed tablets with varying geometries to achieve distinct release profiles: (**A**) poly-layered system, (**B**) floating drug delivery system, and (**C**) monolithic sustained system.

Cylindrical designs ensure constant release until complete API absorption, while horn shapes provide a gradual release, making them suitable for long-term therapies. In contrast, inverted horns enable an immediate initial release followed by a slower, sustained release, which is particularly advantageous for medications requiring high initial doses. Three-dimensional printing enables the fabrication of pharmaceuticals with adaptable designs (cylindrical, horn, etc.) and programmable drug release profiles tailored to specific dosage requirements, dosing intervals, and disease severity. For instance, poly-layered tablets (Figure 12) can be created to accommodate diverse formulations and controlled-release kinetics, enhancing treatment efficacy for conditions requiring precise drug levels, such as Parkinson’s disease, treated with pramipexole, or chronic pain, treated with caffeine and nifedipine. Floating drug delivery tablets with reduced density can delay drug release by remaining in the stomach for extended periods, which is beneficial for conditions requiring gradual release, such as hypertension, treated with propranolol. Monolithic sustained tablets allow for the incorporation of multiple active ingredients with modifiable dissolution rates, optimizing therapeutic absorption based on the severity of the disease and individual patient needs, as demonstrated with baclofen, paracetamol, and ibuprofen [71].

These innovations demonstrate the ability of 3D printing to adapt drug release profiles to meet specific therapeutic requirements (Table 2).

Moreover, additional geometric innovations include oval and pearl designs for immediate-release formulations, such as paracetamol printed via SSE, which has 90% immediate release. Multilayer tablet designs showcase staggered dissolution profiles, such as in the caffeine–paracetamol combination printed via FDM [102] where the caffeine dissolves immediately and the paracetamol dissolves later, optimizing therapeutic efficacy [103]. Similarly, tall cylindrical shapes produced via FDM enable extended release of medications like aripiprazole [104], while honeycomb structures support controlled release of gastro-floating tablets containing pregabalin [105] or baclofen [106], ensuring effectiveness for over 24 h.

Beyond the traditional shapes are one-dimensional formats such as thin films loaded with APIs, as in the case of aripiprazole [107], or gummy forms [108] for pediatric and geriatric [109] patients. These innovative formats improve usability and adherence, including in groups of patients with neurological conditions with characteristics such as reduced visual acuity [110] or swallowing difficulties. This expansion of delivery formats highlights the versatility of 3D printing in addressing diverse patient needs.

**Figure 13:**
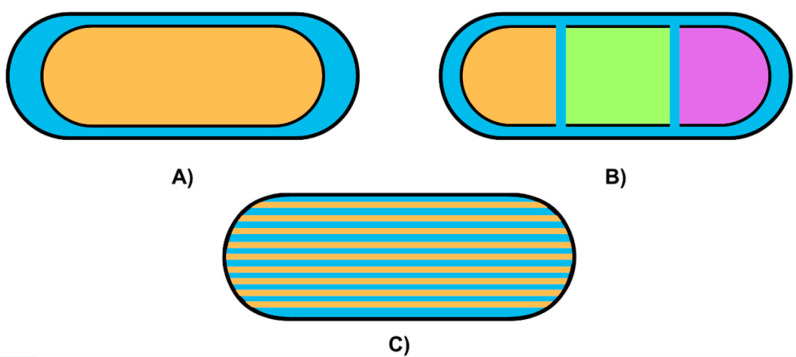
Three-dimensional-printed personalized oral drug delivery systems with varying internal architectures for controlled release: (**A**) core–shell design for encapsulating APIs, (**B**) multi-compartment structure for housing multiple drugs, and (**C**) multilayer configuration for staggered dosage release.

Furthermore, the personalization of oral drug delivery vehicles extends to complex designs featuring multi-compartments. These compartments can house multiple drugs or release a single drug in staggered doses over time (Figure 13), as demonstrated in the case of methylphenidate [82]. By designing compartments with varying thicknesses and volumes, it is possible to create distinct release patterns tailored to specific therapeutic requirements.

The core–shell tablet can be produced using methods like FDM, SSE, SLA, and BJ. FDM deposits materials sequentially, switching filaments to form the core and shell, while SSE extrudes paste-like formulations to create precise layering of the shell around the core. SLA cures layers of photopolymers with high precision, and BJ selectively binds powdered materials to form distinct regions. Similarly, the multi-compartment design can be created using FDM, SSE, and BJ. FDM deposits multiple materials layer by layer, switching filaments to create separate compartments, SSE extrudes paste formulations into specific sections, and BJ selectively binds powdered materials for precise placement of APIs. FDM and SSE are the most practical for versatility, while BJ is ideal for powder-based designs. Furthermore, the multilayered tablet design can be manufactured by FDM, SSE, SLS, SLA, and BJ. FDM alternates filaments for varying compositions, SSE extrudes paste formulations to build alternating drug layers, SLS fuses powdered materials layer by layer for controlled composition, SLA cures photopolymers in precise alternating layers, and BJ selectively deposits powders to form layers with precision. FDM and SSE offer versatility, while SLS and SLA are suited for high-precision designs.

These designs can incorporate liquid, semi-solid, or solid APIs, such as caffeine–paracetamol combinations [111], adding to their flexibility.

Importantly, a key determinant of 3D-printed vehicle performance is the printing angle, which influences both the structural integrity and the release profile of the medication [38]. Factors such as the filament deposition direction relative to the *X*-axis, plot width, and distance between adjacent plots significantly affect the mechanical properties and drug release behavior of the printed oral vehicles [112]. In the production of these oral vehicles, factors like printing path, angle, filament thickness, and mechanical properties are controlled through advanced software and material selection. Precise adjustments in layer patterns, thickness, and infill density directly impact drug release profiles, mechanical strength, and resolution [49]. Material choices, such as tailored polymers or resins, ensure compatibility with the API and desired dissolution rates. These technical considerations underscore the precision required in 3D printing to optimize therapeutic outcomes [113].

Thus, the application of 3D printing in oral drug delivery vehicle design has unlocked unprecedented opportunities for personalization in medicine. By leveraging innovative geometric patterns and compartmentalized structures, it is possible to tailor drug release profiles to specific therapeutic requirements. This level of control and adaptability paves the way for highly effective and patient-friendly oral drug delivery systems, particularly benefiting neurological, pediatric, geriatric, and visually impaired patients, who often require customized medication solutions. For instance, practical examples include colorful gummies designed for pediatric patients, which offer an appealing and playful appearance [114]. For geriatric patients, different textures are incorporated to prevent slipping and ensure ease of swallowing [115]. Moreover, for visually impaired individuals, designs can feature ergonomic grips and integrated Braille printed directly on the medication for improved usability [116]. Together, these examples demonstrate the versatility of 3D printing technology in improving user experience, enhancing accessibility, and addressing the diverse needs of patients across different age groups and abilities.

### 3.5. User Acceptance

Three-dimensional-printed oral medicines have emerged as a promising innovation that is particularly beneficial for treating diseases that affect the CNS, such as Alzheimer’s and Parkinson’s, Attention Deficit Hyperactivity Disorder (ADHD), stroke, epilepsy, and migraines [117,118]. By enabling easier ingestion and superior absorption compared to medicines produced through conventional processes, these oral vehicles offer significant advantages for patients with neurological conditions.

One of the key strengths of 3D-printed medications lies in their ability to be manufactured in various shapes, textures, and flavors, which makes them suitable for patients across all age groups, ranging from neonates [119] and pediatric patients [120] to adults and geriatrics [109]. Furthermore, for visually impaired individuals, tactile medication forms, such as figures, letters, numbers, or patterns like targets or honeycombs, can be created. This allows for quick and accurate identification while reducing the risk of ingestion errors.

**Figure 14:**
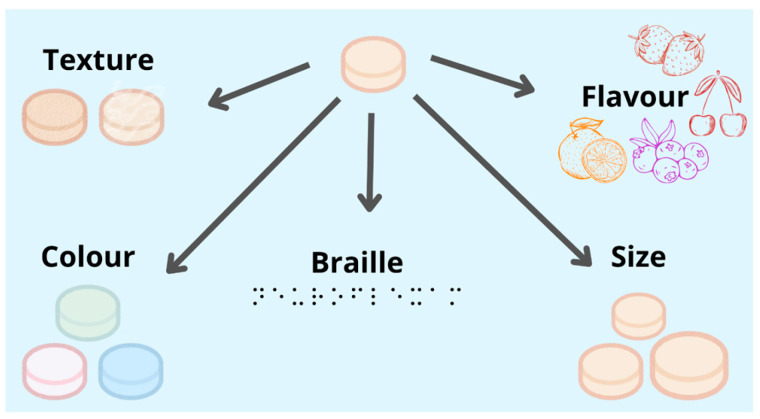
Three-dimensional-printed oral drug delivery vehicles with various parameters for personalized treatment of CNS diseases.

In addition, 3D-printed oral vehicles containing APIs for CNS diseases provide personalized drug dosages [89], addressing the specific needs and preferences of neurological patients [51]. These medications can be tailored to have diverse geometric shapes with customized structural behaviors, various consistencies [27], attractive colors [121], and improved palatability (Figure 14) [45]. For example, jellybean-like consistencies, visually appealing colors such as in purple melatonin, and flavors that enhance the taste of medications like lamotrigine contribute significantly to better adherence to neurological treatments, which is often required for chronic use. Furthermore, the addition of Braille directly onto the medication assists visually impaired individuals in identifying their medicines, enhancing usability, safety, and adherence to treatments [116].

By combining customizable drug dosages, unique shapes and textures, and enhanced visual and taste appeal, 3D-printed oral medicines address the challenges faced by diverse patient populations, including the visually impaired and those requiring long-term medication. This ultimately improves treatment adherence and enhances the overall quality of healthcare delivery in CNS-related treatments.

### 3.6. Quality Processes

Three-dimensional printing in pharmaceuticals represents a significant advancement, offering innovative solutions for improving medication effectiveness while adhering to biosafety standards and regulations for the transport, distribution, and release of APIs [122]. By reducing adverse effects, including the probability of intoxication, and enhancing medication precision, this approach has become highly attractive for fabricating medicines [123].

In industrial 3D printing processes, precision in API dosage and controlled-release mechanisms are critical, particularly for managing CNS diseases, where the margin between therapeutic doses, underdosing, overdosing, and poisoning is narrow [10]. To achieve this, strict methodologies, protocols, and tools are implemented to ensure the quality of pharmaceutical oral vehicles [124]. Notably, the FDA has supported research to better understand the interplay between printing properties, materials, and process quality in 3D-printed pharmaceutical products [22]. This effort marked a milestone in 2015 with the approval of Spritam^®^, the first 3D-printed vehicle for levetiracetam, which signified the beginning of the technology’s application in neurological drugs [125]. Since 2017, the FDA has further established guidelines for the 3D printing of medical products and devices [49].

### 3.7. Regulatory Context

Specific guidelines for the 3D printing of oral medications, including those for neurological treatments, are still lacking; they currently fall under general regulatory frameworks. These medications are governed by Chemistry, Manufacturing, and Controls (CMC), Good Manufacturing Practices (GMP) [126], and international standards set by organizations like Identification of Medicinal Products of the International Organization of Standardization (ISO-IDMP) [127] and the American National Standards Institute (ANSI), which ensure the quality of methods such as FDM, SSE, SLA, DLP, SLS, and BJ for producing oral vehicles for human use [128]. To obtain FDA and EMA approval, it is important to overcome challenges associated to the 3D printing process including those relating to the creation of multilayers, the type of materials used, the interaction between the printed oral vehicle and APIs, and factors affecting the optimal temporal and spatial release of medications [103,129]. The FDA and EMA regulate both the shape and color of 3D-printed oral formulations to ensure safety, quality, and efficacy. The shape of these formulations, such as tablets, capsules, or multi-compartment designs, is assessed for its impact on drug release rates and patient compliance. Regarding color, approved colorants must be safe and non-toxic, and not affect drug efficacy. The FDA allows synthetic dyes like FD&C Red No. 40, Yellow No. 5, and Blue No. 1, as well as natural colorants like carmine and beta-carotene. The EMA authorizes colorants under E-numbers, including E120 (carmine) and E172 (iron oxides). Some colorants, like tartrazine, require allergy warnings, and substances like titanium dioxide are facing increasing restrictions [120]. Additionally, the quality of printers, associated costs, and the acceptance of 3D-printed products by end users are vital factors. In this context, the principal issues that 3D printing faces relate to cost, time, and scale-up.

Currently, various 3D printing methods for producing oral vehicles containing APIs for CNS treatments are under approval at the Center for Drug Evaluation and Research (CDER) in the USA [130]. The FDA continues to investigate these pharmaceutical products, aligning them with global regulations and GMP standards for oral medications, including those used for managing neurological diseases [131].

As research progresses, the focus has shifted toward personalized treatments tailored to specific needs, such as those of children, adults, and elderly patients with altered states of consciousness or visual impairments, aiming to improve treatment adherence and outcomes while advancing the potential of 3D-printed pharmaceutical products in enhancing healthcare delivery.

## 4. Conclusions

The advancements in 3D printing technologies for oral drug delivery in the treatment of CNS diseases, such as Alzheimer’s, Parkinson’s, and epilepsy, offer promising solutions for creating personalized and effective treatments. This study identifies key innovations in 3D printing techniques, such as FDM, SSE, SLA, DLP, SLS, and BJ, and highlights the use of materials like PLA, PVA, PCL, ABS, and HIPS, which enable the production of oral medications with complex geometries and tailored drug release profiles.

The data show significant growth in the number of publications on 3D printing for oral drug delivery in CNS diseases, with an increase from 89 in 2019 to 180 by May 2024, a 102% rise. Notably, the 2024 data cover only five months, indicating potential for a higher annual total. The United States leads in contributions, followed by the United Kingdom, India, Italy, and China. Among institutions, University College London and its affiliates, Inserm, and Harvard Medical School are key contributors. This growth highlights the increasing importance of this research area and the collaborative global efforts driving innovation.

The insights obtained highlight the growing interest in 3D-printed oral drug delivery systems that use different oral vehicles including pills, polypills, caplets, gel caps, multitablets, orodispersible films, and tablets with external patterns and internal structures containing one or more active substances. FDM has emerged as the leading 3D printing technique for CNS disease treatments as it offers several advantages, such as low cost and consistent active substance distribution, and does not need post-processing. Moreover, PLA and PVA have been identified as the most used materials due to their versatility, which enables the creation of personalized medications with tailored release profiles and compartmentalized designs. By addressing the needs of diverse populations, including pediatric, geriatric, and visually impaired patients, 3D-printed medicines not only enhance treatment adherence but also open new frontiers in personalized healthcare delivery.

However, the continued development of these technologies requires addressing several challenges. One major obstacle is the optimization of 3D printing processes to improve precision, particularly with respect to the high temperatures required in FDM, which may not be suitable for all Active Pharmaceutical Ingredients. Additionally, further research is needed to refine the properties of printing materials and explore new combinations to enhance the effectiveness and safety of 3D-printed oral medications.

Future efforts should focus on enhancing the scalability and cost efficiency of 3D printing technologies to make these personalized therapies more accessible. As these innovations move toward commercial application, efforts must also be directed toward regulatory approval processes, ensuring that 3D-printed drugs meet all necessary biosafety and quality standards. Furthermore, the integration of 3D printing in clinical practice needs to be explored further, with particular attention to patient acceptance and adherence, especially in vulnerable populations such as pediatric and geriatric patients.

The continuous evolution of 3D printing technology holds significant potential to revolutionize oral drug delivery systems, particularly for CNS disorders, ultimately improving treatment outcomes and the quality of life for patients. The obstacles and efforts in refining this technology will be crucial for its successful integration into clinical settings.

## Figures and Tables

**Table 1:** Categories and topics in 3D printing for oral drug delivery.

Category	Topic
3D Printing Techniques	Fused Deposition Modeling (FDM).
	Semi-Solid Extrusion (SSE).
	Stereolithography (SLA).
	Digital Light Processing (DLP).
	Selective Laser Sintering (SLS).
	Binder Jetting (BJ).
Applications	Pills.
	Polypills.
	Caplets.
	Gel Caps.
	Multitablets.
	Orodispersible Films.
	Tablets with one or more API(s).
Materials	Polylactic Acid (PLA).
	Polyvinyl Alcohol (PVA).
	Polycaprolactone (PCL).
	Acrylonitrile Butadiene Styrene (ABS).
	High-Impact Polystyrene (HIPS).
Design Factors	Geometric Pattern for Drug Release Control.
	Multi-Compartment Designs.
	Release Profiles.
User Acceptance	Personalized Drug Dosages.
	Customized Shapes and Textures.
	Improved Adherence.
Quality Processes	Precision in API Dosage.
	Biosafety and Regulations.
	FDA Approval for 3D-Printed Drugs.

**Table 2:** Three-dimensional printing techniques, APIs, and their drug release profiles.

3D Printing Technique	API	Release Profile	Reference
FDM	Levodopa	Extended Release	[30]
	Aripiprazole	Immediate Release	[35]
	Olanzapine	Immediate Release	[38]
	Pregabalin	Immediate Release	[47]
	Carbamazepine	Extended Release	[90]
	Levetiracetam	Immediate Release	[91]
	Theophylline	Immediate Release	[92]
SSE	Levetiracetam	Immediate Release	[43]
	Phenytoin	Immediate Release	[93]
	Gabapentin	Extended Release	[94]
	Mirtazapine	Immediate Release	[95]
SLA	Paracetamol	Extended Release	[96]
	Theophylline	Extended Release	[97]
	Methylphenidate	Extended Release	[98]
	Ibuprofen	Extended Release	[99]
SLS	Levetiracetam	Immediate Release	[22]
	Ondansetron	Immediate Release	[83]
	Paracetamol	Immediate Release	[100]
BJ	Diclofenac	Immediate Release	[100]
	Acetaminophen	Immediate Release	[101]

## Data Availability

Data are contained within the article.

## Abbreviations

**Meaning**	**Abbreviations**	**Meaning**	**Abbreviations**
Central Nervous System	CNS	World Health Organization	WHO
Active Pharmaceutical Ingredient	API	Competitive Technology Intelligence	CTI
Fused Deposition Modeling	FDM	Semi-Solid Extrusion	SSE
Stereolithography	SLA	Digital Light Processing	DLP
Selective Laser Sintering	SLS	Binder Jetting	BJ
Neurological Drug Administration Systems	NDDS	Oral Dispersible Films	ODF
Web of Science	WoS	Computer-Aided Design	CAD
Attention Deficit Hyperactive Disorder	ADHD	Polylactic Acid	PLA
Polyvinyl Alcohol	PVA	Polycaprolactone	PCL
Acrylonitrile Butadiene Styrene	ABS	High-Impact Polystyrene	HIPS
Pressure-Assisted Micro Syringes	PAM	Ultraviolet	UV
Hot Melt Extrusion	HME	Center for Drug Evaluation and Research	CDER
Chemistry Manufacturing, and Controls	CMC	Good Manufacturing Practices	GMP
International Organization for Standardization	ISO	American National Standards Institute	ANSI
European Medicines Agency	EMA	International Council for Harmonization	ICH
